# In the national epidemiological bulletins – a selection from recent issues

**DOI:** 10.2807/1560-7917.ES.2018.23.13.180329-1

**Published:** 2018-03-29

**Authors:** Eurosurveillance editorial team

**Affiliations:** 1 European Centre for Disease Prevention and Control (ECDC), Stockholm, Sweden

**Keywords:** infectious diseases, public health, Europe

**Healthcare-associated infections and antibiotic resistance**

 

Characteristics, frequency and distribution of MRSA in Germany - Update 2015/2016

Epidemiologisches Bulletin 5, 2018

1 February 2018, Germany

https://www.rki.de/DE/Content/Infekt/EpidBull/Archiv/2018/Ausgaben/05_18.pdf?__blob=publicationFile

 

**Food- and waterborne diseases** 

 

Human botulism in France, 2013-2016

Bulletin épidémiologique hebdomadaire 3

6 February 2018, France

http://invs.santepubliquefrance.fr/beh/2018/3/pdf/2018_3.pdf

 

Estimates of food-related morbidity and mortality in metropolitan France, 2008-2013

Bulletin épidémiologique hebdomadaire 1

9 January 2018, France

http://invs.santepubliquefrance.fr/beh/2018/1/pdf/2018_1.pdf

 

Gastroenteritis in the autumn of 2017

EPI-ICE, Volume 11(1) 

January 2018, Iceland

https://www.landlaeknir.is/servlet/file/store93/item34358/EPI-ICE_januar_2018.pdf

 

*Plesiomonas shigelloides* after swimming in freshwater

Infectieziekten Bulletin 2018; 29(1)

January 2018, the Netherlands

https://magazines.rivm.nl/2018/01/infectieziekten-bulletin/plesiomonas-shigelloides-na-zwemmen-zoetwater

 

**Hepatitides**

 

Acute and Chronic Hepatitis B 2017

EPI-NEWS 10, 2018

7 March 2018, Denmark

https://www.ssi.dk/Aktuelt/Nyhedsbreve/EPI-NYT/2018/Uge%2010%20-%202018.aspx

 

Ten first years of hepatitis A surveillance through mandatory notification, France, 2006-2015

Bulletin épidémiologique hebdomadaire 5

6 March 2018, France

http://invs.santepubliquefrance.fr/beh/2018/5/pdf/2018_5.pdf

 

**Vaccine-preventable diseases**

 

Vaccine coverage estimates for the school based meningococcal ACWY (MenACWY) adolescent vaccination programme in England, to 31 August 2017

Health Protection Report; 12(3)

27 January 2018, United Kingdom

https://www.gov.uk/government/uploads/system/uploads/attachment_data/file/677839/hpr0318_men-acwy-schl.pdf

 

Vaccine coverage estimates for the school based tetanus, diphtheria and polio (Td/IPV, ‘school leaver booster’) adolescent vaccination programme in England, to 31 August 2017

Health Protection Report; 12(3)

27 January 2018, United Kingdom

https://www.gov.uk/government/uploads/system/uploads/attachment_data/file/677274/hpr0318_tdipv.pdf

 

Measles outbreak 2017 - update 

Epi-Insight 2018;19(1)

January 2018, Ireland

http://ndsc.newsweaver.ie/epiinsight/ovifbvm1o6r?a=1&p=52792563&t=17517774

 

**Respiratory diseases** 

 

Special issue: World Tuberculosis Day, 24 March 2018

Bulletin épidémiologique hebdomadaire, 6-7

20 March 2018, France

http://invs.santepubliquefrance.fr/beh/2018/6-7/pdf/2018_6-7.pdf

 

Group A streptococcal infections: seasonal activity, 2017/18: second report

Health Protection Report; 12(9)

9 March 2018, United Kingdom

https://www.gov.uk/government/uploads/system/uploads/attachment_data/file/687260/hpr0918_sf-gas-scnd.pdf

 

Tuberculosis in a nursing home for the elderly: Use of a interferon-gamma release assay for the screening strategy around one case

Bulletin épidémiologique hebdomadaire, 4

20 February 2018, France

http://invs.santepubliquefrance.fr/beh/2018/4/pdf/2018_4.pdf

 

Tuberculosis 2016

EPI-NEWS, 2018

17 January 2018, Denmark

https://www.ssi.dk/Aktuelt/Nyhedsbreve/EPI-NYT/2018/Uge%203%20-%202018.aspx

 

Legionella risks in mist devices in supermarkets

Infectieziekten Bulletin 2018; 29(1)

January 2018, the Netherlands

https://magazines.rivm.nl/2018/01/infectieziekten-bulletin/legionellarisicos-bij-mistapparaten-nederlandse-supermarkten-0

 

**Sexually transmitted diseases**

 

Sexually transmitted diseases still on the increase

EPI-ICE, Volume 11(1) 

January 2018, Iceland

https://www.landlaeknir.is/servlet/file/store93/item34358/EPI-ICE_januar_2018.pdf

****

**Zoonoses and vector-borne diseases**

 

Rabies 2016 and 2017 and new recommendations for rabies prophylaxis

EPI-NEWS 9, 2018

28 February 2018, Denmark

https://www.ssi.dk/Aktuelt/Nyhedsbreve/EPI-NYT/2018/Uge%209%20-%202018.aspx

 

Common animal associated infections quarterly report (England and Wales): fourth quarter 2017

Health Protection Report; 12(5)

9 February 2018, United Kingdom

https://www.gov.uk/government/uploads/system/uploads/attachment_data/file/680710/hpr0518_zoos.pdf

 

Puumala virus infections and the seed production of trees

Infectieziekten Bulletin 2018; 29(2)

February 2018, the Netherlands

https://magazines.rivm.nl/2018/02/infectieziekten-bulletin/puumalavirusinfecties-en-de-zaadproductie-van-bomen

 

The status of zoonotic diseases in 2016

Infectieziekten Bulletin 2017; 28(10)

14 December 2017, the Netherlands

https://www.rivm.nl/Documenten_en_publicaties/Algemeen_Actueel/Uitgaven/Infectieziekten_Bulletin/Jaargang_28_2017/December/inhoud_december_2017/De_Staat_van_Zo_nosen_in_2016

 

**Other**

****

Reporting of clusters of staphylococcal toxic shock syndrome linked to menstruation, Pays de la Loire (France), 2013 and 2016

Bulletin épidémiologique hebdomadaire, 2

23 January 2018, France

http://invs.santepubliquefrance.fr/beh/2018/2/pdf/2018_2.pdf

